# Predicting Frailty Trajectories Using Interpretable Machine Learning Among Older Adults Following Hip Surgery: Prospective Longitudinal Study

**DOI:** 10.2196/90705

**Published:** 2026-06-16

**Authors:** Jingying Huang, Qinqin Fan, Huiqin Shi, Jiaojiao Chen, Hongbo Gao, Kailai Gu, Jin Yang

**Affiliations:** 1 Sir Run Run Shaw Hospital, No. 3, Qingchun East Road, Hangzhou, Zhejiang, China, 86 17757173794; 2 Shanghai Maritime University, Shanghai, China; 3 Zhejiang Chinese Medical University, Hangzhou, China

**Keywords:** frailty trajectory, hip surgery, predictive model, Extreme Gradient Boosting, XGBoost, Shapley Additive Explanations, SHAP analysis, frailty, aging, older adults

## Abstract

**Background:**

Postoperative frailty is highly prevalent among older adults undergoing hip surgery and is closely linked to poor clinical outcomes. Despite growing interest in understanding its progression, the temporal patterns of frailty remain underexplored. Moreover, there is a lack of validated models that can predict frailty trajectories and stratify patients by risk in the early postoperative period.

**Objective:**

This study aimed to identify distinct frailty trajectories within 6 months following hip surgery in older adults and to explore their associated predictors. An interpretable machine-learning model was developed and internally validated for individualized risk prediction and was implemented as a clinically accessible web-based calculator.

**Methods:**

This prospective longitudinal observational study was conducted among older adults undergoing hip surgery at a tertiary hospital in China. Frailty assessments were performed preoperatively and at 1, 3, and 6 months postoperatively. A total of 209 participants who completed the 6-month follow-up were included in the analysis. Frailty was assessed using the Frailty Index, and group-based trajectory modeling was applied to identify distinct frailty progression patterns. Predictive variables were selected using the least absolute shrinkage and selection operator regression. An interpretable Extreme Gradient Boosting (XGBoost) model was developed using a 60:40 training-test data split. Model performance was evaluated in terms of discrimination, calibration, and clinical utility. Interpretability was assessed using SHAP (Shapley Additive Explanations) at both the global and individual levels.

**Results:**

Three distinct frailty trajectories were identified: low-fluctuation frailty (55/209, 26%), high-improvement frailty (81/209, 39%), and high-deterioration frailty (73/209, 35%). Twelve predictors grounded in the Health Ecology Model were selected, spanning individual characteristics, interpersonal networks, and the living environment. The XGBoost model demonstrated excellent discrimination, with a microaverage area under the receiver operating characteristic curve of 0.98 (95% CI 0.96‐0.99) in the training set and 0.93 (95% CI 0.90‐0.96) in the test set. Calibration was acceptable, with a weighted Brier score of 0.0852. Decision curve analysis showed favorable clinical utility across a range of threshold probabilities. A web-based risk calculator was developed to facilitate personalized frailty trajectory prediction.

**Conclusions:**

The XGBoost model demonstrated strong predictive performance and interpretability, enabling the early identification of older patients at risk for adverse frailty trajectories following hip surgery. This tool may support targeted interventions and improve perioperative care in geriatric populations.

## Introduction

Population aging has emerged as a significant global public health challenge. With shifting demographic structures, the incidence of orthopedic conditions among older adults has risen substantially. In 2000, there were an estimated 1.6 million hip fracture cases worldwide, a number projected to increase to 6.3 million by 2050 [1]. Hip osteoarthritis is also common in older adults, with an incidence of up to 10%, and its end-stage form leads to significant pain and disability, severely impacting quality of life [1]. For conditions such as hip fractures and advanced hip osteoarthritis, hip surgery remains the primary treatment, effectively alleviating pain, restoring joint function, and improving patient outcomes [2]. Notably, approximately 85% of patients undergoing hip surgery are aged 60 years or older and often present with multimorbidity and frailty [3].

Frailty is an age-related clinical syndrome characterized by a progressive decline in physiological function and multisystem reserve, which impairs the ability to withstand stressors, such as illness, trauma, or surgery, and increases vulnerability to adverse outcomes [4,5]. A growing body of evidence has identified frailty as an independent predictor of poor postoperative outcomes, including falls, functional decline, rehospitalization, and mortality [6-8]. Among older patients undergoing surgery, the prevalence of frailty ranges from 41.8% to 50.3% and may reach up to 65% in those undergoing hip surgery [9,10]. This elevated prevalence reflects the dual impact of both the underlying health status and surgical stress. On the one hand, hip fracture itself is often associated with frailty-related conditions such as sarcopenia, impaired balance, and malnutrition. On the other hand, perioperative factors such as prolonged bed rest, pain, and reduced mobility can accelerate muscle loss and disrupt homeostasis, further depleting physiological reserves through endocrine, metabolic, and immune dysregulation [11,12]. Common postoperative complications, including delirium and infection, may further delay recovery and exacerbate functional deterioration. Even 1 year after surgery, frailty remains present in 25.6% to 27.7% of patients [13].

Importantly, frailty is increasingly recognized as a dynamic and potentially reversible condition. Its core principle emphasizes that early identification and proactive intervention can delay or even reverse its progression [14]. The distinct phases of postoperative recovery, rehabilitation, and stabilization provide a critical window to observe changes in frailty status. These trajectories are shaped by factors such as baseline reserve, perioperative complications, and psychosocial conditions. While some patients recover steadily, others experience worsening frailty and increased dependency [15]. Studies suggest that frailty trajectory patterns better predict outcomes than static measurements, with rapid deterioration or high variability indicating increased risk [16]. These findings underscore the need for tools that can identify frailty trajectories early and guide intervention timing. While previous studies have examined frailty trajectories in various disease contexts, only a few have developed predictive models [17,18]. These models are typically restricted to specific populations, rely on a narrow set of variables, and employ conventional statistical approaches, limiting their robustness and generalizability. Furthermore, static frailty assessment tools, such as the Clinical Frailty Scale and the FRAIL Scale, fail to capture the multidimensional, nonlinear nature of frailty progression. Therefore, there is a pressing need for predictive models that integrate multilevel risk factors and offer strong interpretability.

The Health Ecology Model (HEM) offers a comprehensive framework for viewing health as the outcome of interactions among individual, behavioral, and environmental factors [19]. It emphasizes that health is determined by personal characteristics (eg, age, sex, body weight, psychological status, and lifestyle), as well as by social support, living conditions, economic status, and broader policy environments. This perspective aligns with the multifactorial etiology of frailty, which is shaped by both intrinsic and extrinsic factors [18,20]. Methodologically, Extreme Gradient Boosting (XGBoost) is a robust ensemble learning algorithm well-suited for modeling high-dimensional, nonlinear data and performs reliably even with relatively small sample sizes. SHAP (Shapley Additive Explanations), grounded in cooperative game theory, enhances the interpretability of machine-learning models by quantifying the global and individual-level contributions of each feature to prediction outcomes [21].

Therefore, guided by the HEM, this study integrates predictors across individual, behavioral, and environmental domains to construct an interpretable XGBoost-based predictive model for frailty trajectories within 6 months following hip surgery in older patients. SHAP is used to visualize and interpret the contribution of key predictors, and a web-based calculator is developed for clinical applications. This study aims to support the stratification of patients into distinct frailty trajectory subgroups, enabling more precise screening, targeted intervention, and optimized allocation of health care resources.

## Methods

### Study Design

This was a prospective, longitudinal cohort study, reported in accordance with the 2024 TRIPOD + AI (Transparent Reporting of a Multivariable Prediction Model for Individual Prognosis or Diagnosis—Artificial Intelligence) guidelines for the development and validation of multivariable prediction models [22].

### Population

This study employed convenience sampling to recruit older adults who underwent hip surgery under general anesthesia at a tertiary hospital in China between March 2024 and March 2025. Eligible patients were followed prospectively for 6 months postoperatively, with follow-up assessments conducted from March 2024 to September 2025.

Inclusion criteria were age 60 years or older; scheduled for elective hip surgery, including total hip arthroplasty, hemiarthroplasty, or internal fixation; and sufficient communication ability to complete study assessments. Exclusion criteria were severe psychiatric disorders that prevented adequate cooperation; substantial hearing or visual impairments that precluded completion of questionnaires; palliative status (ie, life expectancy <6  mo); or current enrollment in other studies likely to affect frailty outcomes. Participants were withdrawn if they were lost to follow-up due to relocation or hospital transfer, voluntarily withdrew consent, or died of causes unrelated to the study protocol.

Baseline data and preoperative frailty assessments were collected through face-to-face interviews conducted by trained researchers. Follow-up assessments at 1, 3, and 6 months postoperatively were conducted via structured telephone interviews.

### Sample Size

As there is currently no universally accepted sample size calculation method for trajectory-based multiclass machine-learning prediction models, the sample size was pragmatically estimated using a conventional epidemiological formula to ensure adequate baseline cohort representativeness. The required sample size was calculated as follows [23]: $n={\left(\frac{Z}{\delta }\right)}^{2}\hat{\phi }(1-\hat{\phi })$, where *Z* is the critical value at a 95% CI, $\overset{\wedge }{\phi }$ is the estimated incidence of frailty, and *δ* is the acceptable margin of error. Based on a pilot study indicating a 6-month postoperative frailty incidence of 14% and setting *d* at 5%, the calculated sample size was 185. After accounting for a 10% attrition rate, the final target sample size was set at no fewer than 206 cases to ensure the epidemiological representativeness of the baseline cohort.

### Ethical Considerations

Ethical approval for the study was obtained from the Ethics Committee of Sir Run Run Shaw Hospital, Zhejiang University School of Medicine (number 2024-2687-01). Informed consent was obtained from all participants before the study commenced.

### Outcomes Definition

Frailty was assessed preoperatively and at 1, 3, and 6 months postoperatively using a modified Frailty Index (FI) based on the cumulative deficit model developed by Rockwood and Mitnitski [24]. Tailored to the clinical characteristics of older adults undergoing hip surgery, we developed a 37-item population-specific FI covering 6 domains: chronic diseases, mental health, activities of daily living (ADLs), cognitive function, and symptoms/signs. Although several predictive variables share conceptual domains with components of the FI, none of the specific indicators used to construct the FI were directly included as predictors in the model. FI scores ranged from 0 to 1, with higher scores indicating greater frailty. A score <0.25 was classified as nonfrail and a score ≥0.25 was classified as frail [25]. The full list of FI items is provided in Multimedia Appendix 1.

### Candidate Predictor Variables

Based on a review of the literature and expert consultation, potential risk factors were explored within the HEM, encompassing individual characteristics, behavioral and psychological factors, interpersonal networks, and the living environment. Key individual characteristics included demographic and socioeconomic factors (eg, gender, marital status, education level, and income), physiological indicators (high-sensitivity C-reactive protein [hsCRP]), health behaviors (smoking and drinking), and clinical history (number of comorbidities and surgery type). Functional status was assessed using the Barthel Index for ADLs, with scores indicating varying levels of independence [26]. Nutritional status was evaluated via the Mini Nutritional Assessment–Short Form [27], with scores categorizing participants as malnourished, at risk, or well-nourished.

Behavioral and psychological factors, including depressive symptoms, were measured using the 5-item Geriatric Depression Scale, while sleep quality was assessed using the Pittsburgh Sleep Quality Index. For interpersonal networks, family function was assessed using the Family APGAR (Adaptability, Partnership, Growth, Affection, Resolve) Index [28], and social support was measured using the Social Support Rating Scale. Finally, environmental factors were evaluated using a self-developed Living Environment Questionnaire (LEQ), which assesses home adaptability, perceived environmental burden, community resource accessibility, and residential satisfaction. The LEQ demonstrated strong content validity and was tailored to the needs of older adults. Detailed descriptions of these variables and the respective scales are provided in Multimedia Appendix 1.

### Statistical Analysis

A complete-case analysis was conducted. Missing data primarily arose from loss to follow-up during the longitudinal assessments, and participants with incomplete follow-up data were excluded from the analysis. To assess the missing data mechanism, the Little test for missing completely at random (MCAR) was performed. In addition, baseline characteristics between participants included in the final analysis and those who were lost to follow-up were compared to evaluate the potential for selection bias. Categorical variables are expressed as frequencies and percentages. Differences between the training and test sets were assessed using the chi-square test. Continuous variables with a normal distribution are presented as mean (SD), while nonnormally distributed variables are shown as median (IQR). Depending on the data distribution, the *t* test or the Mann-Whitney *U* test was applied.

Group-based trajectory modeling was utilized to identify frailty trajectories in older patients with hip fractures. The optimal model was selected based on clinical relevance, fit indices, and classification quality, with a preference for simpler models with fewer groups. Model fit was evaluated using the following criteria [29]: (1) Bayesian Information Criterion (BIC): lower values indicate a better fit; (2) average posterior probability (AvePP): AvePP greater than 0.7 for all groups, with the smallest group proportion exceeding 5%; (3) odds of correct classification (OCC): OCC was greater than 5; (4) relative entropy (E_k_): E_k_ was higher than 0.8.

The data were randomly split into training and test sets in a 60:40 ratio using stratified sampling to preserve the original distribution of the 3 frailty trajectory classes across the dataset. To prevent overfitting, LASSO (least absolute shrinkage and selection operator) regression was applied to the training set for high-dimensional feature selection. The L1 regularization term enabled simultaneous variable selection and coefficient shrinkage. Model performance was evaluated using 10-fold cross-validation, with the optimal penalty parameter (λ) selected according to the 1 SE rule to ensure model parsimony and generalizability. To evaluate potential overlap and multicollinearity among predictors, variance inflation factors (VIFs) were calculated for variables retained after LASSO selection. A VIF <5 was considered to indicate no significant multicollinearity [30].

The selected features were used to construct the XGBoost model. Because no trajectory class represented an extremely small proportion of the sample, no additional class imbalance handling strategies (eg, oversampling or SMOTE [Synthetic Minority Oversampling Technique]) were applied during model training. To improve model robustness and reduce overfitting, regularization and subsampling strategies were incorporated into the XGBoost model. During training, a 3-fold inner cross-validation was employed to estimate performance. For the test set, the area under the receiver operating characteristic curve, accuracy, *F*_1_-score, sensitivity, and specificity were computed. The calibration curve was used to compare predicted probabilities with actual outcomes across frailty trajectories. Decision curve analysis was performed to assess net clinical benefit. Finally, SHAP values were used to interpret the XGBoost model, ranking feature importance based on the mean absolute SHAP values. The XGBoost model was deployed on a cloud platform [31] using the “Shiny” package, creating an online prediction calculator for frailty trajectories following hip surgery.

Statistical analysis and graphical representations were performed using IBM SPSS 26.0, R 4.5.1 (R Core Team), and Python 3.9 (Python Software Foundation), with the “traj,” “glmnet,” “xgboost,” and “shap” packages. All statistical tests were 2-sided, and statistical significance was set at α=.05.

## Results

### Characteristics of Participants

A total of 300 patients were screened for the study, of whom 78 were excluded due to severe hearing impairment, significant visual decline, communication barriers, or other factors. Four patients with incomplete data were also excluded, leaving 218 patients enrolled. Multimedia Appendix 2 presents the flowchart of the participants included in this study. During the follow-up period, 9 participants were lost to follow-up at 6 months, yielding an attrition rate of 4.1%. Given that frailty trajectories were identified using group-based trajectory modeling based on 3 repeated follow-up assessments, participants with incomplete longitudinal data were excluded to ensure the stability and interpretability of trajectory classification. To evaluate the missing data mechanism, we conducted a missing data analysis (Multimedia Appendix 2). The Little MCAR test indicated that the missing data were consistent with the assumption of MCAR (*χ*²_4_=3.750, *P*=.44). In addition, no significant differences were observed in baseline characteristics between participants retained in the analysis and those lost to follow-up (Multimedia Appendix 2), suggesting a low risk of selection bias.

### Frailty Prevalence and Trajectories

Frailty levels among older patients undergoing hip surgery exhibited a dynamic trend, with an initial increase followed by a gradual decline. The prevalence of frailty was 70.81% (148/209) before surgery, peaked at 84.21% (176/209) at 1 month postoperatively, and subsequently decreased to 63.16% (132/209) at 3 months and 45.45% (95/209) at 6 months.

A 3-class trajectory model demonstrated the best fit based on BIC, AvePP, and E_k_ values (Multimedia Appendix 3). It showed the lowest BIC change (59.36). All classes had AvePP values greater than 0.7 and OCC values exceeding 5. The E_k_ was 0.905, indicating high classification precision. Therefore, the 3-trajectory model was selected to characterize postoperative frailty trajectories among older adults undergoing hip surgery. Based on the estimated intercepts and slopes, trajectory 0 was labeled as low-fluctuation frailty, comprising 55 out of 209 individuals (26%), with a lower baseline frailty level, a modest increase at 1 month, and a subsequent decline. Trajectory 1 was labeled as high-improvement frailty, including 81 out of 209 (39%) individuals who had a higher baseline frailty level and experienced continuous functional recovery. Trajectory 2 was labeled as high-deterioration frailty, consisting of 73 out of 209 (35%) individuals who started with a high frailty level and demonstrated progressive worsening over time. Detailed parameter estimates and visual representations are provided in Multimedia Appendix 3. Given the distinct clinical patterns observed among the 3 classes, a multiclass modeling approach was employed to support the subsequent predictive analysis.

### Predictor Variable Selection

A total of 209 participants were randomly assigned to a training set (n=126) and a testing set (n=83) in a 60:40 ratio. The training set was used for model development, while the testing set was reserved for independent performance evaluation. Baseline demographic and clinical characteristics were compared between the 2 sets, with statistically significant differences observed only in marital status and type of medical insurance (Table 1).

**Table 1.** Comparison of baseline characteristics between the training and test sets.

Variables	Total (N=209)	Training set (n=126)	Test set (n=83)	*χ*^2^ (*df*) or *Z* value	*P* value
Age (y), median (IQR)	74 (68-83)	75 (68-83)	74 (68-82)	−3.343^a^	.73
Gender, n (%)				0.720 (1)^b^	.40
Male	66 (31.6)	37 (29.4)	29 (34.9)		
Female	143 (68.4)	89 (70.6)	54 (65.1)		
Marital status, n (%)				6.326 (1)^b^	.01
With spouse	58 (27.8)	27 (21.4)	31 (37.3)		
Without spouse	151 (72.2)	99 (78.6)	52 (62.7)		
Education level, n (%)				3.023 (3)^b^	.39
Primary or below	135 (64.6)	79 (62.7)	56 (67.5)		
Middle school	47 (22.5)	33 (26.2)	14 (16.9)		
High school	20 (9.6)	10 (7.9)	10 (12)		
College or above	7 (3.3)	4 (3.2)	3 (3.6)		
Type of work, n (%)				2.574 (2)^b^	.28
Manual labor	125 (59.8)	71 (56.3)	54 (65.1)		
Mental labor	64 (30.6)	40 (31.7)	24 (28.9)		
Unemployed or other	20 (9.6)	15 (11.9)	5 (6.0)		
Residence place, n (%)				1.669 (1)^b^	.20
Rural	117 (56.0)	66 (52.4)	51 (61.4)		
Urban	92 (44.0)	60 (47.6)	32 (38.6)		
Live arrangement, n (%)				1.752 (1)^b^	.19
Alone	41 (19.6)	21 (16.7)	20 (24.1)		
With family members	168 (80.4)	105 (83.3)	63 (75.9)		
Monthly income (￥), n (%)				0.182 (2)^b^	.91
<1000	68 (32.5)	42 (33.3)	26 (31.3)		
1000~5000	107 (51.2)	63 (50.0)	44 (53.0)		
>5000	34 (16.3)	21 (16.7)	13 (15.7)		
Medical insurance, n (%)				3.873 (1)^b^	.049
Yes	10 (4.8)	6 (4.3)	4 (1.2)		
No	199 (95.2)	117 (92.9)	82 (98.8)		
BMI (kg/m^2^), n (%)				0.515 (2)^b^	.77
<18.5	24 (11.5)	15 (11.9)	9 (10.8)		
18.5~24	113 (54.1)	70 (55.6)	43 (51.8)		
>24	72 (34.4)	41 (32.5)	31 (37.3)		
Albumin (g/L), n (%)				0.430 (1)^b^	.51
<40	138 (66.0)	81 (64.3)	57 (68.7)		
≥40	71 (34.0)	45 (35.7)	26 (31.3)		
hsCRP^c^ (mg/L), n (%)				0.160 (1)^b^	.69
≥6	130 (62.2)	77 (61.1)	53 (63.9)		
<6	79 (37.8)	49 (38.9)	30 (36.1)		
Smoking status, n (%)				4.117 (2)^b^	.13
Current smoker	14 (6.7)	5 (4.0)	9 (10.8)		
Never smoker	172 (82.3)	108 (85.7)	64 (77.1)		
Former smoker	23 (11.0)	13 (10.3)	10 (12.0)		
Drinking status, n (%)				2.194 (2)^b^	.33
Current drinking	36 (17.2)	18 (14.3)	18 (21.7)		
Never drinking	160 (76.6)	99 (78.6)	61 (73.5)		
Former drinking	13 (6.2)	9 (7.1)	4 (4.8)		
Number of teeth, median (IQR)	14 (8-18)	14 (8-18)	14 (8-18)	−0.079^a^	.94
Number of comorbidities, n (%)				1.352 (1)^b^	.25
≥2	101 (48.3)	65 (51.6)	36 (43.4)		
<2	108 (51.7)	61 (48.4)	47 (56.6)		
Surgery type, n (%)				0.947 (2)^b^	.62
Hip internal fixation	58 (27.8)	32 (25.4)	26 (31.1)		
Total hip arthroplasty	88 (42.1)	54 (42.9)	34 (41.0)		
Hemiarthroplasty	63 (30.1)	40 (31.7)	23 (27.7)		
Social activity frequency, n (%)				2.046 (1)^b^	.15
≥3 times per week	147 (70.3)	84 (66.7)	63 (75.9)		
<3 times per week	62 (29.7)	42 (33.3)	20 (24.1)		
Activities of daily living, n (%)				3.997 (3)^b^	.26
Complete dependence	6 (2.9)	5 (4.0)	1 (1.2)		
Severe dependence	46 (22.0)	25 (19.8)	21 (25.3)		
Moderate dependence	120 (57.4)	77 (61.1)	43 (51.8)		
Mild dependence	37 (17.7)	19 (15.1)	18 (21.7)		
Nutritional status, n (%)				1.565 (2)^b^	.46
Malnourished	36 (17.2)	25 (19.8)	11 (13.3)		
At risk	64 (30.6)	38 (30.2)	26 (31.3)		
Normal	109 (52.2)	63 (50.0)	46 (55.4)		
Depressive, n (%)				0.512 (1)^b^	.47
Yes	19 (9.1)	10 (7.9)	9 (10.8)		
No	190 (90.9)	116 (92.1)	74 (89.2)		
Sleep quality, n (%)				0.013 (1)^b^	.91
Poor	74 (35.4)	45 (35.7)	29 (34.9)		
Good	135 (64.6)	81 (64.3)	54 (65.1)		
Family function, n (%)				2.221 (2)^b^	.33
Severe dysfunction	62 (29.7)	40 (31.7)	22 (26.5)		
Moderate dysfunction	74 (35.4)	47 (37.3)	27 (32.5)		
Good family functioning	73 (34.9)	39 (31.0)	34 (41.0)		
Social support level, median (IQR)	30 (27-34)	30 (27-33)	30 (27-34)	−0.218^a^	.83
Living environment score, median (IQR)	12 (11-14)	12 (11-14)	12 (11-14)	−0.243^a^	.81

a Mann-Whitney *U* test.

b Chi-square test.

c hsCRP: high-sensitivity C-reactive protein.

**Figure 1.**
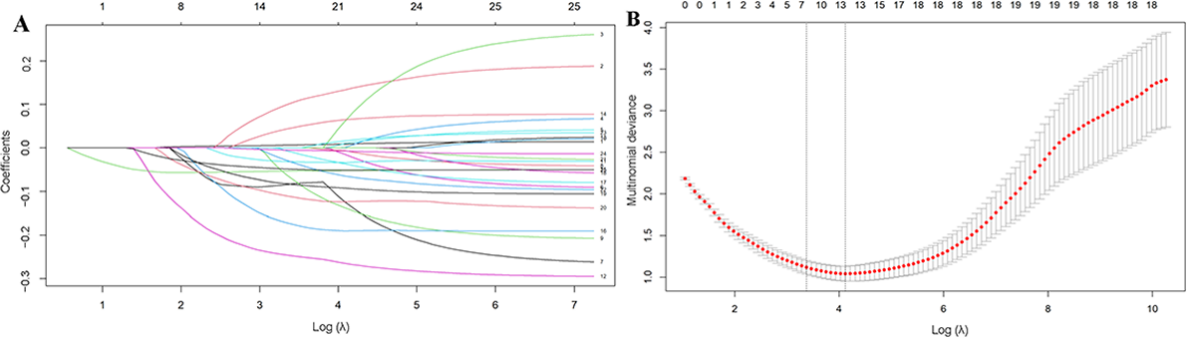
Feature selection using the LASSO (least absolute shrinkage and selection operator) regression model. (A) LASSO coefficient profiles of the 25 candidate predictors. (B) Selection of the optimal tuning parameter (λ) using 10-fold cross-validation.

Twenty-five candidate predictors were entered into the LASSO regression for feature selection. Using the 1 SE criterion (λ=0.0342), 12 variables were retained, including age, gender, living arrangement, albumin, hsCRP, drinking status, teeth number, comorbidities number, ADLs, nutritional status, social support level, and living environment. The feature selection process is illustrated in Figure 1, with additional details provided in Multimedia Appendix 4. Multicollinearity diagnostics showed that the VIF values of the retained predictors ranged from 1.07 to 1.67, with all values below 5, indicating no evidence of significant multicollinearity. Detailed results are presented in Multimedia Appendix 4. These variables were subsequently incorporated into the XGBoost model to predict postoperative frailty trajectories and to explore the relative contribution of each predictor to the classification outcomes.

### Model Development and Evaluation

**Figure 2.**
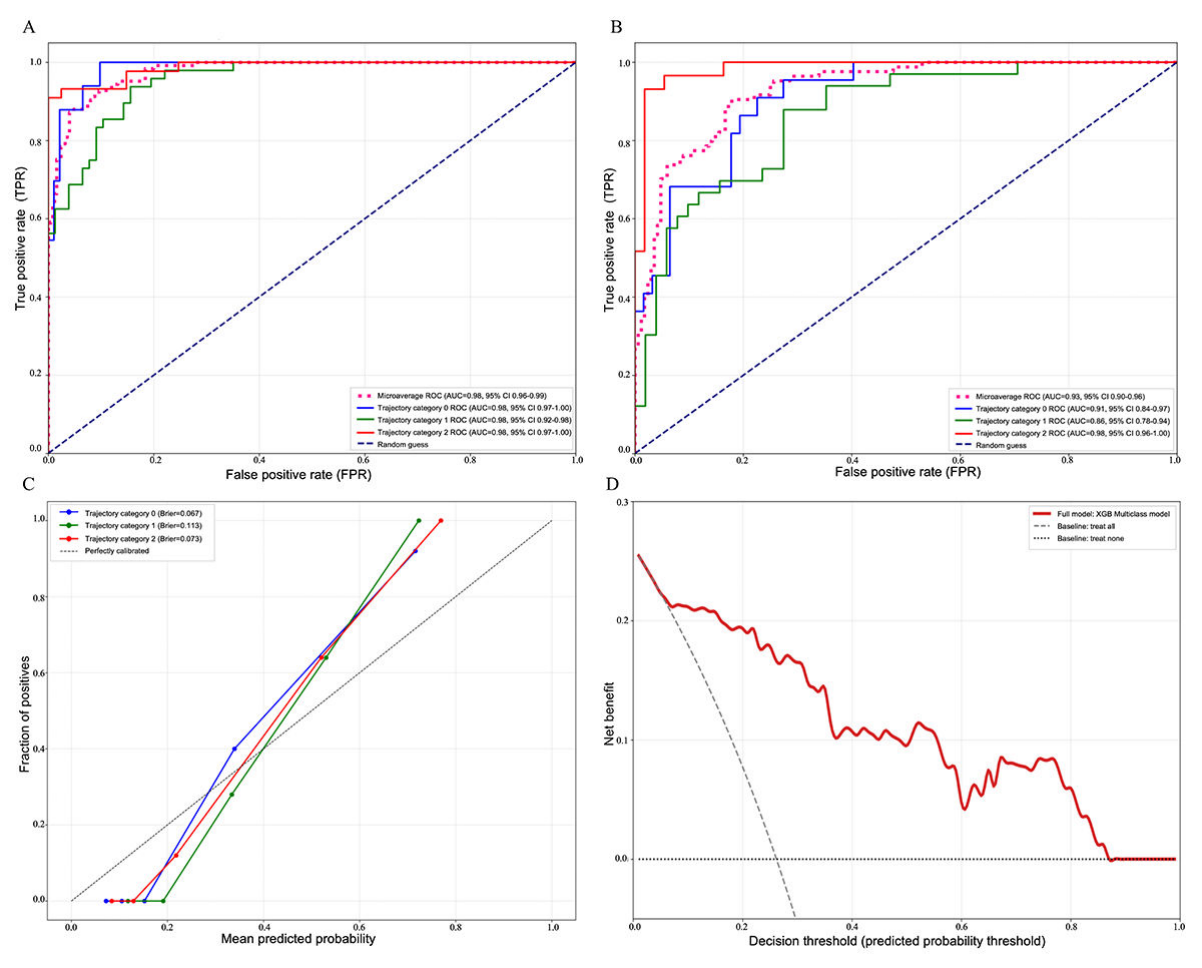
Predictive performance and clinical utility of the frailty trajectory models. (A) Receiver operating characteristic (ROC) curves for the training set. (B) ROC curves for the test set. (C) Calibration plot of the Extreme Gradient Boosting (XGBoost) model. (D) Decision curve analysis (DCA) of the XGBoost model. AUC: area under the ROC curve. Dashed and dotted lines indicate the strategies of treating “all” or “no” patients, respectively.

To optimize XGBoost performance, key hyperparameters were tuned using grid search and cross-validation to improve model generalizability and accuracy. The model achieved excellent discriminative performance, with microaverage area under the receiver operating characteristic curve of 0.98 (95% CI 0.96‐0.99) in the training set (Figure 2A) and 0.93 (95% CI 0.90‐0.96) in the testing set (Figure 2B). Additional metrics, including accuracy, *F*_1_-score, sensitivity, and specificity, are presented in Table 2. Calibration curves (Figure 2C) closely approximated the 45° line, indicating good agreement between predicted and observed probabilities, with a weighted Brier score of 0.085. Decision curve analysis demonstrated consistently greater net clinical benefit across a range of threshold probabilities compared to the “treat-all” and “treat-none” strategies, supporting the model’s potential for clinical applications (Figure 2D).

**Table 2.** The values of the evaluation metrics for the multiclass trajectory model in the test set.

Trajectory model	AUC^a^ (95% CI)	Accuracy (95% CI)	*F*_1_-score (95% CI)	Sensitivity (95% CI)	Specificity (95% CI)
Frailty low fluctuation	0.91 (0.84-0.96)	0.88 (0.82-0.93)	0.87 (0.81-0.92)	0.88 (0.82-0.93)	0.88 (0.82-0.93)
Frailty high improvement	0.86 (0.77-0.94)	0.85 (0.78-0.91)	0.83 (0.76-0.89)	0.80 (0.72-0.87)	0.85 (0.78-0.91)
Frailty high deterioration	0.98 (0.96-1.00)	0.96 (0.93-0.99)	0.97 (0.94-1.00)	0.95 (0.92-0.98)	0.98 (0.95-1.00)
Overall	0.93 (0.90-0.96)	0.89 (0.84-0.94)	0.89 (0.83-0.94)	0.87 (0.80-0.93)	0.90 (0.85-0.95)

a AUC: area under the receiver operating characteristic curve.

### Model Interpretation

**Figure 3.**
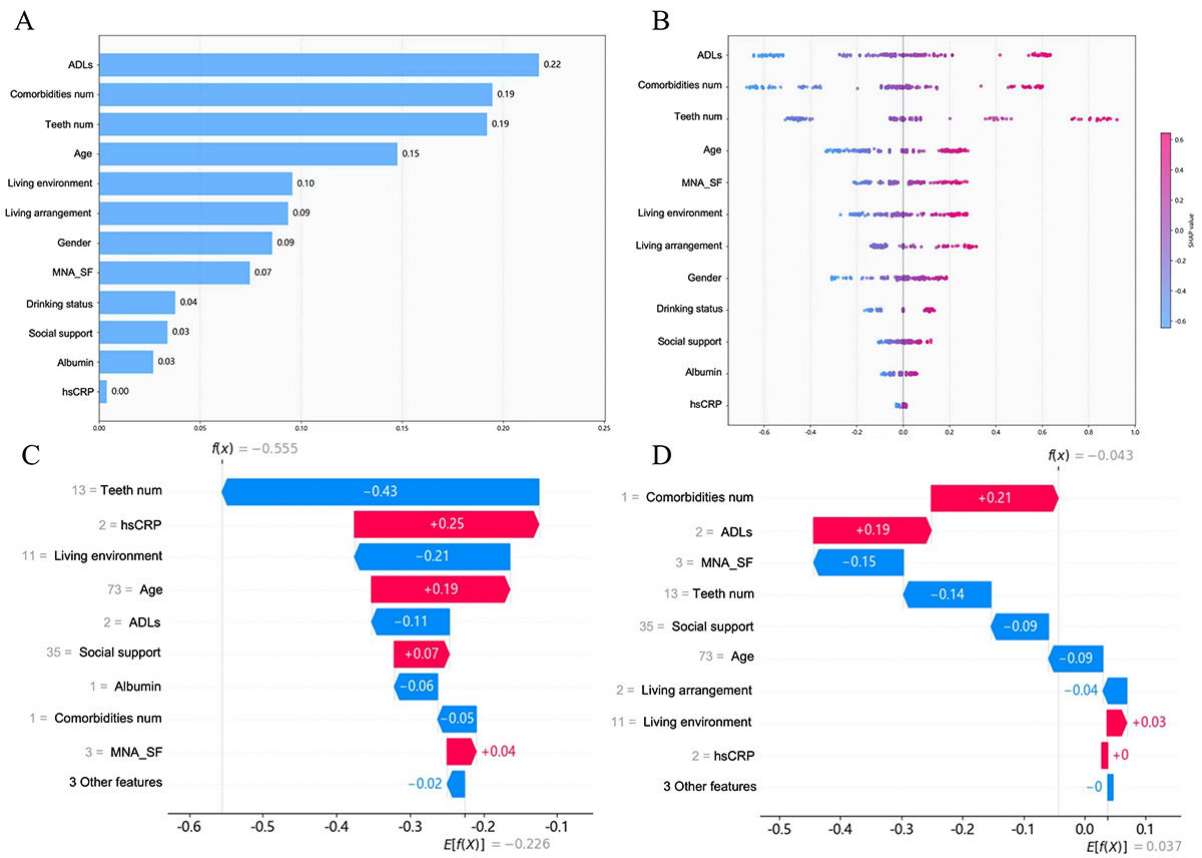
SHAP (Shapley Additive Explanations)–based interpretation of the Extreme Gradient Boosting (XGBoost) frailty trajectory model. (A) Feature importance ranked by mean absolute SHAP values. (B) The beeswarm plot showing the distribution and direction of SHAP values for each feature. (C) The SHAP waterfall plot for an illustrative patient from the low-fluctuation frailty trajectory. (D) The SHAP waterfall plot for an illustrative patient from the high-deterioration frailty trajectory. ADL: activities of daily living; hsCRP: high-sensitivity C-reactive protein; MNA-SF: Mini Nutritional Assessment–Short Form.

The SHAP algorithm was used to visualize and interpret the contribution of each predictor to the frailty trajectory model. As shown in Figure 3A, the bar plot displays the mean SHAP values of the 12 selected predictors, reflecting their average impact on the model output. ADLs had the greatest predictive importance, followed by the number of comorbidities and the number of teeth. Figure 3B presents the SHAP value distributions for each feature, with color gradients from blue (low values) to red (high values) representing the magnitude of each predictor and its influence on individual predictions.

To visualize case-specific model explanations, Figure 3C and D presents SHAP waterfall plots for illustrative patients from the low-fluctuation and high-deterioration frailty trajectories, respectively. For these samples, the predicted model outputs were −0.555 and −0.043, compared with baseline values of −0.226 and 0.037. For the low-fluctuation trajectory, favorable living environments and higher teeth counts showed strong negative contributions to the model output, offsetting the positive contributions of age and hsCRP. In contrast, the high-deterioration trajectory was primarily characterized by positive contributions from comorbidities and ADL limitations, whereas nutritional status, social support, dental health, and age contributed negatively to the model output.

**Figure 4.**
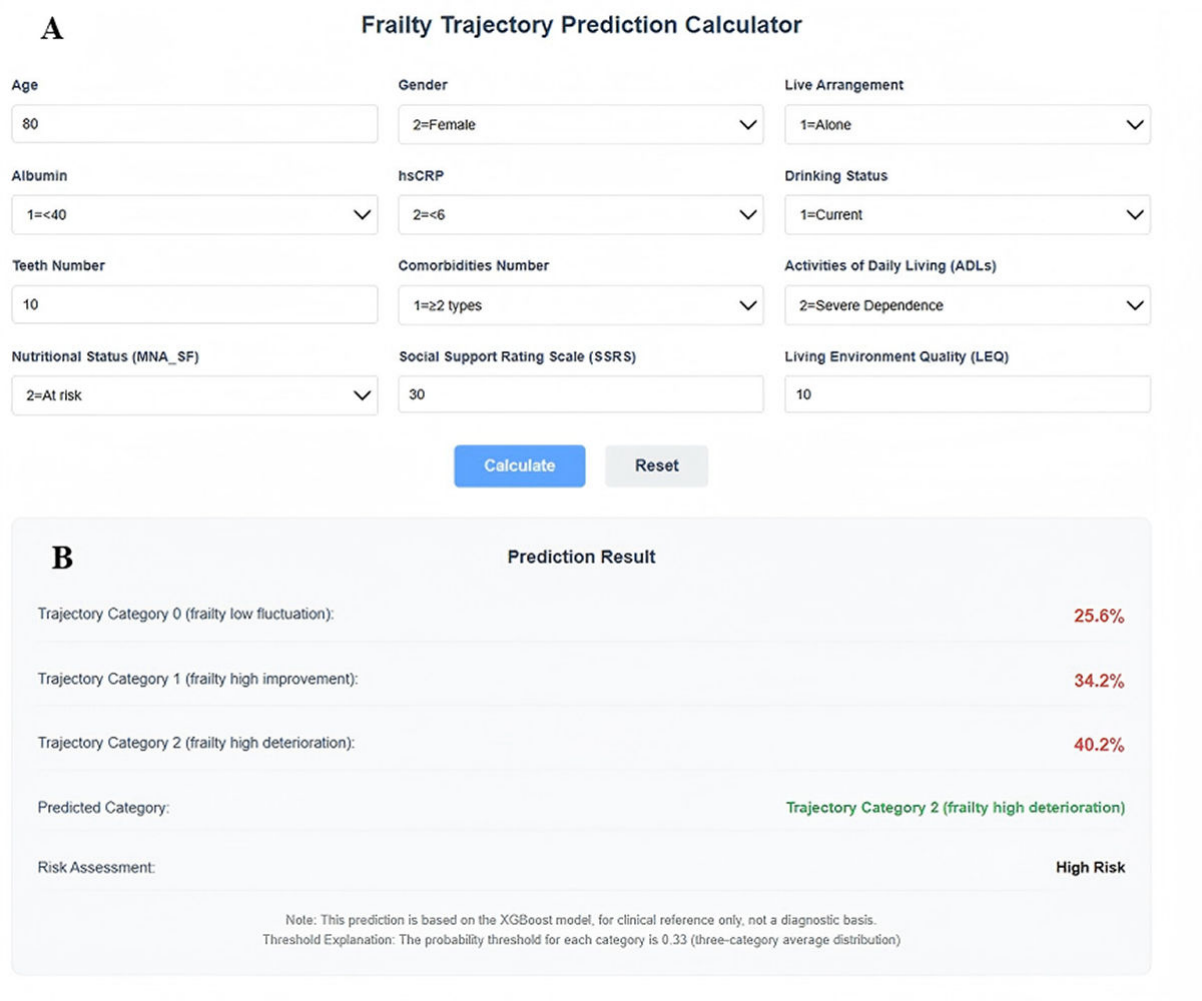
(A) Web-based calculator for predicting the probability of frailty trajectories following hip surgery. (B) Prediction result. hsCRP: high-sensitivity C-reactive protein; MNA-SF: Mini Nutritional Assessment–Short Form.

A web-based frailty trajectory prediction calculator was developed and is freely available online [32]. The user interface of the application is shown in Figure 4. This tool allows users to input key patient characteristics and obtain estimated probabilities for each frailty trajectory class, which may support frailty risk assessment and individualized management planning.

## Discussion

### Principal Findings

This study identified 3 distinct postoperative frailty trajectories among older adults following hip surgery: low fluctuation, high improvement, and high deterioration. These findings highlight that frailty after hip surgery is a dynamic process with substantial interindividual variability rather than a static condition. By moving beyond traditional binary outcomes [17,18], this trajectory-based approach provides a more nuanced understanding of recovery patterns that are often obscured in aggregate analyses. Furthermore, by integrating multidimensional predictors within the HEM, we developed a prediction model that demonstrated good discrimination and calibration performance. Collectively, these findings provide a basis for early risk stratification and the development of targeted, individualized intervention strategies in postoperative care.

Although the overall prevalence of frailty followed a rise-then-fall trajectory over the 6-month postoperative period, consistent with prior studies such as Huang et al [33], this general trend concealed the diversity of individual recovery paths. The early peak in frailty may be attributed to systemic inflammatory responses, catabolic stress, and functional limitations induced by postoperative pain, while the subsequent decline likely reflects recovery mechanisms, including tissue healing, pain relief, and rehabilitation engagement. Trajectory modeling in this study revealed 3 distinct frailty patterns: low fluctuation, high improvement, and high deterioration. These heterogeneous trajectories suggest that despite experiencing the same surgical stressor, older patients vary substantially in their recovery due to differences in baseline physiological reserve, comorbid conditions, social support, and environmental context.

A closer examination of these subgroups further highlights their clinical relevance. The low fluctuation group, despite having a relatively low baseline frailty level, experienced a notable increase at 1 month postoperatively. This transient deterioration may indicate reduced physiological resilience. Prior studies suggest that abrupt fluctuations in frailty may signal underlying instability and predict adverse events such as falls or rehospitalization more accurately than static frailty status [34]. For this group, interventions should aim to maintain functional stability by implementing prehabilitation, early mobilization, multimodal pain control, and prevention of iatrogenic complications [12]. In contrast, the high-improvement group demonstrated a steady recovery trajectory, likely reflecting reversible functional impairments driven primarily by hip pathology. These patients may benefit from standard rehabilitation care without intensive resource input, allowing health care systems to prioritize those at greater risk. The high-deterioration group represents a clinically vulnerable subgroup with persistent decline, suggesting unresolved physiological or psychosocial deficits. Comprehensive geriatric assessments and tailored multidisciplinary interventions are warranted for this population, ideally guided by frameworks such as the World Health Organization (WHO)’s ICOPE (Integrated Care for Older People), which emphasizes multidomain support targeting mobility, cognition, nutrition, sensory function, and social engagement [35,36].

Based on the identified frailty trajectories, we developed a predictive model to support early risk identification and targeted intervention. Guided by the HEM, the model incorporated variables spanning individual characteristics, interpersonal networks, and living environment. Although some key predictors (eg, ADLs, comorbidity burden, and nutritional status) are conceptually related to domains of the FI, the multicollinearity analysis did not indicate significant statistical overlap among the predictors. This suggests that, despite conceptual relatedness, these variables do not represent redundant information from a statistical perspective. Nevertheless, these factors may partly reflect baseline vulnerability. Therefore, the model should not be interpreted as predicting frailty trajectory class membership entirely independent of baseline frailty severity. Instead, it is more appropriately viewed as an early risk stratification tool that integrates multidimensional information. Consistent with this interpretation, the SHAP analysis identified ADLs, number of comorbidities, number of teeth, age, and living environment as the top contributors to the model predictions.

In line with previous research [17,37], this study confirmed that impaired ADLs and a greater number of comorbidities were the strongest predictors of frailty deterioration. These variables represent fundamental aspects of functional dependence and chronic disease burden. Declining ADLs reflect a depletion of multisystem physiological reserves and may initiate a downward cycle in which the reduced function limits physical activity, weakens muscle strength, and accelerates frailty progression [37]. Similarly, multimorbidity contributes through persistent low-grade inflammation, polypharmacy, and cumulative organ damage, ultimately undermining mobility and physiological stability [38]. For individuals with impaired ADLs and multiple comorbidities, the early implementation of interventions such as individualized rehabilitation, optimized management of chronic conditions, and comprehensive medication review may be essential to interrupt this adverse trajectory [39,40]. In addition, age and teeth number were identified as important predictors. While age reflects irreversible biological decline, tooth loss serves as a modifiable marker of oral frailty. Reduced dentition in older adults impairs chewing function and nutritional intake, which in turn promotes sarcopenia and accelerates frailty [41]. These findings highlight the importance of incorporating oral health screening into perioperative management, along with timely interventions such as denture repair, chewing function training, and personalized nutrition support [14].

Among the identified predictors, living environment was the only environmental factor ranked among the top 5, highlighting its important role in frailty progression. Although the LEQ was a self-developed instrument, its core domains are conceptually aligned with the WHO age-friendly environment framework [42]. Previous studies have shown that environmental factors such as housing safety, community resources, social participation, and perceived environmental support are closely associated with frailty and its progression in older adults [43-45]. Notably, our findings demonstrated that living environment independently predicted frailty trajectories beyond individual-level characteristics. This finding is consistent with recent evidence showing that more positive perceptions of community environments are associated with lower frailty risk [46], as well as the findings of Mak et al [47], who reported sustained effects of environmental factors on frailty progression. For older adults undergoing hip surgery, environmental barriers after discharge may limit rehabilitation adherence and increase the risks of falls and readmission, thereby accelerating frailty progression. These findings emphasize the need to extend clinical care beyond hospital settings by incorporating environmental risk assessments into discharge planning and transitional care. Practical strategies may include predischarge home safety evaluations, caregiver education, and community linkage interventions to foster a more supportive recovery environment and reduce frailty-related risks [48].

In addition to the core predictors, the model identified several supplementary variables, including living arrangement, gender, nutritional status, drinking behavior, social support, albumin, and hsCRP. Although these variables contributed less individually, they enriched the understanding of frailty trajectories from physiological, behavioral, and demographic perspectives. These results indicate the importance of combining model-based risk identification with comprehensive biopsychosocial assessments to detect individualized frailty risk profiles and guide targeted interventions. Building on this, implementing a Fracture Liaison Service (FLS) may offer an effective integrative care pathway [49]. Through its structured components of assessment, education, intervention, and follow-up, Fracture Liaison Service enables continuous, coordinated management for older adults with hip fractures, supporting the transition from early risk prediction to precise clinical action.

### Limitations

This study has several limitations. First, it was a prospective single-center study with a relatively small sample size, focusing exclusively on older patients undergoing elective hip surgery under general anesthesia, which may limit the generalizability of the findings to broader populations such as individuals receiving emergency surgery, regional anesthesia, or in other age groups. In addition, the relatively limited sample size for multiclass machine-learning modeling may increase the risk of overfitting and unstable feature selection despite the use of LASSO-based dimensionality reduction and internal cross-validation. Therefore, both the current prediction model and the associated web-based calculator should be considered exploratory and proof-of-concept tools rather than clinically deployable systems. Prospective multicenter external validation, calibration assessment, and health-economic evaluation are warranted before routine clinical integration.

Second, a complete case analysis was used to handle missing data. Although the Little MCAR test suggested a completely-at-random mechanism and no baseline differences were observed between retained and excluded participants, departures from this assumption cannot be ruled out. If missingness were related to the severity of frailty or the presence of comorbidities, this could introduce bias and potentially affect trajectory classification and model performance. Therefore, the findings should be interpreted with caution. Future studies may consider more robust approaches for handling missing data, where appropriate, to further assess the robustness of the results.

Third, although the model incorporated multilevel predictors guided by the socioecological framework, some important but difficult-to-measure confounders, such as genetic predisposition or deeper physiological reserves, may have been missed. Future studies could leverage technologies such as wearable devices and multiomics approaches to improve measurement precision and expand variable inclusion. Finally, the 6-month follow-up period may capture only early or acute frailty changes and fail to reflect long-term trajectories. Longer follow-up and analytical methods such as competing risk models may help differentiate between temporary functional decline and persistent frailty, thus identifying critical intervention windows.

### Conclusion

This study developed and internally validated a proof-of-concept machine-learning model to predict frailty trajectories within 6 months after hip surgery in older adults. Grounded in the HEM, the model integrated multidimensional predictors and showed good performance in discrimination, calibration, and clinical utility. Key contributing factors included functional status, comorbidity burden, oral health, age, and living environment. The model was translated into a web-based tool to support early identification and personalized frailty management; it currently serves as a foundational step. Comprehensive external validation across diverse clinical settings is strictly required before this tool can be deployed in routine clinical practice. These findings provide methodological and practical support for shifting perioperative care toward a proactive and individualized approach.

## Supplementary material

10.2196/90705 Multimedia Appendix 1 Variables and measurements.

10.2196/90705 Multimedia Appendix 2 Participant characteristics and missing data.

10.2196/90705 Multimedia Appendix 3 Frailty trajectory modeling (group-based trajectory modeling).

10.2196/90705 Multimedia Appendix 4 Predictor selection and multicollinearity.

## Abbreviations

ADL: activity of daily living
APGAR: Adaptability, Partnership, Growth, Affection, Resolve
AvePP: average posterior probability
BIC: Bayesian Information Criterion
FI: Frailty Index
HEM: Health Ecology Model
ICOPE: Integrated Care for Older People
LASSO: least absolute shrinkage and selection operator
LEQ: Living Environment Questionnaire
MCAR: missing completely at random
OCC: odds of correct classification
SHAP: Shapley Additive Explanations
SMOTE: Synthetic Minority Oversampling Technique
TRIPOD+AI: Transparent Reporting of a Multivariable Prediction Model for Individual Prognosis or Diagnosis—Artificial Intelligence
WHO: World Health Organization
XGBoost: Extreme Gradient Boosting
