# Supplementary material for: Predicting Frailty Trajectories Using Interpretable Machine Learning Among Older Adults Following Hip Surgery: Prospective Longitudinal Study
Source: JMIR Aging. 2026 Jun 16;9:e90705. doi: 10.2196/90705 (PMC13271583; doi:10.2196/90705)
Supplement: Multimedia Appendix 1 [file aging-v9-e90705-s001.docx]

**Table S1: Frailty Index (FI)**

Instructions: The following questions are used to assess your level of frailty. Please tick “√” according to your actual condition. Frailty Index Calculation: FI Score = Total number of deficits / 37

| No. | Item | Scoring |
| --- | --- | --- |
| 1 | Heart disease | □ Yes = 1  □ No = 0 |
| 2 | Liver disease | □ Yes = 1  □ No = 0 |
| 3 | Stroke or cerebrovascular disease | □ Yes = 1  □ No = 0 |
| 4 | Malignant tumor | □ Yes = 1  □ No = 0 |
| 5 | Chronic lung disease (e.g., chronic bronchitis, emphysema) | □ Yes = 1  □ No = 0 |
| 6 | Chronic kidney disease | □ Yes = 1  □ No = 0 |
| 7 | Stomach or gastrointestinal disease | □ Yes = 1  □ No = 0 |
| 8 | Parkinson’s disease | □ Yes = 1  □ No = 0 |
| 9 | Arthritis or rheumatism | □ Yes = 1  □ No = 0 |
| 10 | Feeling fatigued when doing things (past week) | □ Always = 1  □ Occasionally = 0.5  □ Rarely = 0 |
| 11 | Feeling lonely (past week) | □ Always = 1  □ Occasionally = 0.5  □ Rarely = 0 |
| 12 | Feeling fearful (past week) | □ Always = 1  □ Occasionally = 0.5  □ Rarely = 0 |
| 13 | Difficulty concentrating on tasks | □ Always = 1  □ Occasionally = 0.5  □ Rarely = 0 |
| 14 | Needs help with shopping | □ Yes = 1  □ No = 0 |
| 15 | Needs help with housework | □ Yes = 1  □ No = 0 |
| 16 | Needs help with cooking | □ Yes = 1  □ No = 0 |
| 17 | Needs help taking medication | □ Yes = 1  □ No = 0 |
| 18 | Needs help managing money | □ Yes = 1  □ No = 0 |
| 19 | Needs help bathing | □ Yes = 1  □ No = 0 |
| 20 | Needs help dressing | □ Yes = 1  □ No = 0 |
| 21 | Needs help transferring (e.g., bed to chair) | □ Yes = 1  □ No = 0 |
| 22 | Needs help eating | □ Yes = 1  □ No = 0 |
| 23 | Needs help using the toilet | □ Yes = 1  □ No = 0 |
| 24 | Needs help climbing stairs | □ Yes = 1  □ No = 0 |
| 25 | Urinary incontinence | □ Yes = 1  □ No = 0 |
| 26 | Fecal incontinence | □ Yes = 1  □ No = 0 |
| 27 | Able to walk 1 km | □ Yes = 0  □ No = 1 |
| 28 | Able to walk 100 m | □ Yes = 0  □ No = 1 |
| 29 | Able to bend, kneel, or squat | □ Yes = 0  □ No = 1 |
| 30 | Able to lift 5 kg | □ Yes = 0  □ No = 1 |
| 31 | Able to pick up a coin from the table | □ Yes = 0  □ No = 1 |
| 32 | Vision difficulties | □ Yes = 1  □ No = 0 |
| 33 | Hearing difficulties | □ Yes = 1  □ No = 0 |
| 34 | Self-rated health status | □ Very poor = 1  □ Poor = 0.75  □ Fair = 0.5  □ Good = 0.25   □ Excellent = 0 |
| 35 | Does not know today’s date | □ Yes = 1  □ No = 0 |
| 36 | Does not know home address | □ Yes = 1  □ No = 0 |
| 37 | Memory decline | □ Yes = 1  □ No = 0 |

**TEXT: Candidate predictor variables**

Based on a review of the literature and expert consultation, potential risk factors were explored from the following dimensions within the HEM^[1]^: individual characteristics, behavioral and psychological factors, interpersonal networks, and living environment .

***Individual characteristics***

1. **Comprehensive profile of individual attributes**

This dimension included demographic and socioeconomic variables, including gender, marital status, education level, occupation, place of residence, living arrangement, monthly income, and medical insurance type; physiological indicators, including body mass index, albumin, C-reactive protein, and teeth number; health-related behaviors and clinical history, including smoking, drinking, number of comorbidities, and surgery type; as well as social activity and engagement, measured by the frequency and diversity of participation in activities over the past month, such as interacting with neighbors or friends, playing games, exercising, joining community events, volunteering, or attending educational programs.

1. **Activities of daily living**

The Barthel Index (BI), originally introduced by Mahoney et al. ^[2]^, is a widely used scale that assesses activities of daily living (ADLs) across 10 domains, including feeding, bathing, dressing, toileting, continence, mobility, and stair climbing. Total scores range from 0 to 100, with higher scores indicating greater independence. A score of 100 denotes full independence; 75-95 indicates mild dependence; 50-70, moderate; 25-45, severe; and <20, completely dependence. The BI has demonstrated strong test-retest and inter-rater reliability, with test-retest and inter-rater coefficients of 0.89 and 0.95, respectively.

**③ Nutritional status**

Participants’ nutritional status was assessed using the Mini Nutritional Assessment-Short Form (MNA-SF) ^[3]^, a validated tool for evaluating nutrition in older adults across diverse clinical and community settings. The scale comprises six items, yielding a total score from 0 to 14. Scores of 0-7 indicate malnutrition, 8-11 suggest a risk of malnutrition, and scores ≥12 reflect normal nutritional status. The internal consistency of the MNA-SF was acceptable, with a Cronbach’s alpha of 0.832.

***Behavioral and psychological***

**①** **Depressive symptoms.**

Depressive symptoms were assessed using the five-item Geriatric Depression Scale (GDS-5), a brief screening tool developed by Hoyl et al.^[4]^ based on the original GDS. Each dichotomous item is scored 0 or 1, yielding a total score ranging from 0 to 5. A score of ≥2 indicates possible depression, with higher scores reflecting greater symptom severity. The tool has demonstrated good psychometric properties, with a sensitivity of 0.94, specificity of 0.81, and inter-rater reliability of 0.88.

**② Sleep quality**

Sleep quality was evaluated using the Pittsburgh Sleep Quality Index (PSQI), a widely used instrument for assessing subjective sleep over the past month^[5]^. The PSQI comprises 19 self-rated and 5 observer-rated items, grouped into seven components: sleep latency, sleep duration, sleep disturbances, subjective sleep quality, use of sleep medication, sleep efficiency, and daytime dysfunction. Each component is rated on a 0-3 scale, with a global score ranging from 0 to 21. Scores <7 suggest good sleep quality, while scores ≥7 indicate poor sleep quality.

***Interpersonal networks***

1. **Family function**

The Family APGAR Index, developed by Smilkstein et al. ^[6]^, was used to assess satisfaction with family functioning across five dimensions: adaptability, partnership, growth, affection, and resolve. Each item was rated on a 3-point Likert scale, with higher scores indicating better family support. Total scores of 0-3 suggest severe dysfunction, 4-6 indicate moderate dysfunction, and 7-10 reflect good family functioning. In this study, the Cronbach’s alpha coefficient was 0.890, demonstrating high internal consistency.

1. **Social support**

To assess perceived social support, the Social Support Rating Scale (SSRS), developed by Xiao Shuiyuan ^[7]^, was used in this study. The 10-item instrument encompasses three dimensions: subjective support (4 items), objective support (3 items), and support utilization (3 items). Higher scores indicate stronger perceived social support. The SSRS reported a test-retest coefficient of 0.92 and Cronbach’s alpha values ranging from 0.89 to 0.94.

***Living environment***

A self-developed Living Environment Questionnaire (LEQ) was used to assess environmental factors influencing frailty progression. Informed by findings from existing literature, the 10-item tool covers four domains: (1) home adaptability (e.g., elevator access, bathroom safety), (2) perceived environmental burden (e.g., noise, lighting, ventilation), (3) community resource accessibility (e.g., proximity to pharmacies, transportation, fitness and health facilities), and (4) subjective residential satisfaction. Each item is rated on a 3-point Likert scale (0-2), with total scores ranging from 0 to 20; higher scores indicate more supportive environments for maintaining independence. The LEQ was reviewed by five experts in geriatrics and rehabilitation. Content validity was acceptable, with I-CVI ranging from 0.83 to 1.00 and S-CVI of 0.93. Pilot testing confirmed its feasibility and clarity among older adults.
