# Supplementary material for: Predicting Frailty Trajectories Using Interpretable Machine Learning Among Older Adults Following Hip Surgery: Prospective Longitudinal Study
Source: JMIR Aging. 2026 Jun 16;9:e90705. doi: 10.2196/90705 (PMC13271583; doi:10.2196/90705)
Supplement: Multimedia Appendix 2 [file aging-v9-e90705-s002.docx]

**Figure S1. Flow chart of the participants included in this study**

| **Table S2 Missing data analysis** | | | | | | | |
| --- | --- | --- | --- | --- | --- | --- | --- |
|  | **N** | **Mean** | **Std. Deviation** | **Missing** | | **No. of Extremes^a^** | |
|  |  |  |  | **Count** | **Percent** | **Low** | **High** |
| Age | 218 | 75.15 | 9.086 | 0 | .0 | 0 | 0 |
| Number of teeth | 218 | 13.21 | 6.584 | 0 | .0 | 0 | 0 |
| Social support level | 218 | 30.65 | 6.222 | 0 | .0 | 1 | 8 |
| Living environment score | 218 | 12.50 | 2.459 | 0 | .0 | 1 | 0 |
| Gender | 218 |  |  | 0 | .0 |  |  |
| Marital status | 218 |  |  | 0 | .0 |  |  |
| Education level | 218 |  |  | 0 | .0 |  |  |
| Type of work | 218 |  |  | 0 | .0 |  |  |
| Residence place | 218 |  |  | 0 | .0 |  |  |
| Live arrangement | 218 |  |  | 0 | .0 |  |  |
| Monthly income | 218 |  |  | 0 | .0 |  |  |
| Medical insurance | 218 |  |  | 0 | .0 |  |  |
| BMI | 218 |  |  | 0 | .0 |  |  |
| Albumin | 218 |  |  | 0 | .0 |  |  |
| hsCRP | 218 |  |  | 0 | .0 |  |  |
| Smoking status | 218 |  |  | 0 | .0 |  |  |
| Drinking status | 218 |  |  | 0 | .0 |  |  |
| Number of comorbidities | 218 |  |  | 0 | .0 |  |  |
| Surgery type | 218 |  |  | 0 | .0 |  |  |
| Social activity frequence | 218 |  |  | 0 | .0 |  |  |
| Activities of daily living | 218 |  |  | 0 | .0 |  |  |
| Nutritional status | 218 |  |  | 0 | .0 |  |  |
| Depressive | 218 |  |  | 0 | .0 |  |  |
| Sleep quality | 218 |  |  | 0 | .0 |  |  |
| Family support level | 218 |  |  | 0 | .0 |  |  |
| Frailty_trajectory | 209 |  |  | 9 | 4.1 |  |  |

| a. Number of cases outside the range (Q1 - 1.5*IQR, Q3 + 1.5*IQR). |
| --- |

**Table S3 Comparison of baseline characteristics between retained participants and those lost to follow-up**

| Variables | Included Patients (n = 209) | Lost to follow-up (n = 9) | *t/Z/χ^2^*value | *P*-value |
| --- | --- | --- | --- | --- |
| Age (years), median (IQR) | 74 (15) | 74 (11) | -0.273 | 0.783 |
| Gender, n (%) |  |  | 2.259 | 0.155 |
| Male | 66 (31.6) | 5 (55.6) |  |  |
| Female | 143 (68.4) | 4 (44.4) |  |  |
| Marital status, n (%) |  |  | 1.104 | 0.285 |
| With spouse | 59 (28.2) | 4 (44.4) |  |  |
| Without spouse | 150 (71.8) | 5 (55.6) |  |  |
| Education level, n (%) |  |  | 3.524 | 0.243 |
| Primary or below | 135 (64.6) | 4 (44.4) |  |  |
| Middle school | 47 (22.5) | 3 (33.3) |  |  |
| High school | 20 (9.6) | 1 (11.1) |  |  |
| College or above | 7 (3.3) | 1 (11.1) |  |  |
| Type of work, n (%) |  |  | 0.409 | 1.000 |
| Manual labor | 125 (59.8) | 6 (66.7) |  |  |
| Mental labor | 64 (30.6) | 3 (33.3) |  |  |
| Unemployed or Other | 20.0 (9.6) | 0 (0.0) |  |  |
| Residence place, n (%) |  |  | 1.793 | 0.305 |
| Rural | 117 (56.0) | 3 (33.3) |  |  |
| Urban | 92 (44.0) | 6 (66.7) |  |  |
| Live arrangement, n (%) |  |  | 1.008 | 0.390 |
| Alone | 41 (19.6) | 3 (33.3) |  |  |
| With family members | 168 (80.4) | 6 (66.7) |  |  |
| Monthly income, n (%) |  |  | 4.002 | 0.123 |
| ＜1000 | 68 (32.5) | 2 (22.2) |  |  |
| 1000~5000 | 107 (51.2) | 3 (33.3) |  |  |
| ＞5000 | 34 (16.3) | 4 (44.4) |  |  |
| Medical insurance, n (%) |  |  | 0.451 | 1.000 |
| Yes | 10 (4.8) | 0 (0.0) |  |  |
| NO | 199 (95.2) | 9 (100.0) |  |  |
| BMI, median (IQR) | 22.6 (5.2) | 21.3 (3.9) | -1.897 | 0.058 |
| Albumin, mean (SD) | 37.7 (4.4) | 39.2 (3.1) | -0.972 | 0.332 |
| C-reactive protein, median (IQR) | 13.1 (37.7) | 7.4 (9.9) | -1.371 | 0.170 |
| Smoking status, n (%) |  |  | 4.927 | 0.056 |
| Current smoker | 14 (6.7) | 2 (22.2) |  |  |
| Never smoker | 172 (82.3) | 5 (55.6) |  |  |
| Former smoker | 23 (11.0) | 2 (22.2) |  |  |
| Drinking status, n (%) |  |  | 0.936 | 0.657 |
| Current drinking | 36 (17.2) | 1 (11.1) |  |  |
| Never drinking | 160 (76.6) | 7 (77.8) |  |  |
| Former drinking | 13 (6.2) | 1 (11.1) |  |  |
| Number of teeth, median (IQR) | 14 (10) | 14 (11) | -0.357 | 0.721 |
| Number of comorbidities, n (%) |  |  | 0.052 | 1.000 |
| ≥2 | 101 (48.3) | 4 (44.4) |  |  |
| ＜2 | 108 (51.7) | 5 (55.6) |  |  |
| Surgery type, n (%) |  |  | 4.335 | 0.125 |
| Hip internal fixation | 58 (27.8) | 3 (33.3) |  |  |
| Total hip arthroplasty | 88 (42.1) | 6 (66.7) |  |  |
| Hemiarthroplasty | 63 (30.1) | 0 (0.00) |  |  |
| Social activity frequence, n (%) |  |  | 0.893 | 0.459 |
| ≥3 times per week | 147 (70.3) | 5 (55.6) |  |  |
| ＜3 times per week | 62 (29.7) | 4 (44.4) |  |  |
| Activities of daily living, median (IQR) | 65 (23) | 70 (18) | -1.180 | 0.238 |
| Nutritional status, median (IQR) | 12 (4) | 10 (3) | -1.033 | 0.302 |
| Depressive, n (%) |  |  | 1.709 | 0.211 |
| Yes | 19 (9.1) | 2 (22.2) |  |  |
| NO | 190 (90.0) | 7 (77.8) |  |  |
| Sleep quality, n (%) |  |  | 1.516 | 0.290 |
| Poor | 74 (35.4) | 5 (55.6) |  |  |
| Good | 135 (64.6) | 4 (44.4) |  |  |
| Family support level, median (IQR) | 5 (5) | 7 (5) | -1.746 | 0.081 |
| Social support level, median (IQR) | 30 (7) | 33 (15) | -1.692 | 0.091 |
| Living environment score, median (IQR) | 12 (3) | 12 (2) | -0.065 | 0.948 |
