# Supplementary material for: Predicting Frailty Trajectories Using Interpretable Machine Learning Among Older Adults Following Hip Surgery: Prospective Longitudinal Study
Source: JMIR Aging. 2026 Jun 16;9:e90705. doi: 10.2196/90705 (PMC13271583; doi:10.2196/90705)
Supplement: Multimedia Appendix 3 [file aging-v9-e90705-s003.docx]

**Table S4 Performance evaluation metrics for different trajectory models**

| ***Class*** | ***Avepp(%)*** | ***OCC*** | ***P_j_(%)*** | ***π_j_(%)*** | ***BIC*** | ***△BIC*** | ***E_k_*** |
| --- | --- | --- | --- | --- | --- | --- | --- |
| *1C* | 100.00 |  | 100.00 | 100.00 | -2708.98 |  | 0.000 |
| *2C* | 97.41-95.35 | 27.4-28.2 | 57.42-42.58 | 57.91-42.09 | -2526.13 | 182.85 | 0.879 |
| ***3C*** | **96.65-94.23-98.39** | **80.1-26.9-110.4** | **26.32-38.76-34.93** | **26.51-37.82-35.67** | **-2466.77** | **59.36** | **0.905** |
| *4C* | 97.49-93.85-89.95-92.61 | 112.7-45.0-25.8-41.3 | 25.84-25.36-24.88-23.92 | 25.65-25.31-25.74-23.30 | -2443.14 | 23.62 | 0.878 |
| *5C* | 96.94-90.92-93.82-92.64-95.09 | 169.4-77.5-45.6-40.2-61.4 | 15.31-11.96-25.36-22.97-24.40 | 15.75-11.44-24.97-23.85-23.99 | -2404.03 | 39.12 | 0.903 |
| *6C* | 96.87-94.60-92.69-89.44-90.60-95.96 | 169.0-136.3-64.5-30.7-40.5-126.3 | 15.31-11.48-16.27-22.49-19.14-15.31 | 15.49-11.40-16.44-21.64-19.22-15.82 | -2392.86 | 11.17 | 0.893 |
| Note: Avepp, Average Posterior Probability; OCC, Odds of Correct Classification; P_j_ (%), Percentage of Category I;π_j_(%), Proportion of Category i; BIC, Bayesian Information Criterion;  △BIC, Change in BIC**;** E_k_, Relative Entropy. | | | | | | | |

**Table S5 Model fit evaluation for each trajectory group in the optimal model**

| **Group** | **Group** | **Parameter** | ***β*** | ***SE*** | ***t*** | ***P*** |
| --- | --- | --- | --- | --- | --- | --- |
| 3 *Trajectory* | Trajectory 0 | Intercept | 0.10154 | 0.01491 | 6.810 | <0.001 |
|  |  | Linear | 0.18371 | 0.02307 | 7.963 | <0.001 |
|  |  | Quadratic term | -0.06116 | 0.00703 | -8.700 | <0.001 |
|  | Trajectory 1 | Intercept | 0.41502 | 0.01008 | 41.173 | <0.001 |
|  |  | Linear | -0.08675 | 0.00539 | -16.095 | <0.001 |
|  | Trajectory 2 | Intercept | 0.44132 | 0.00969 | 45.544 | <0.001 |
|  |  | Linear | 0.02246 | 0.00546 | 4.114 | <0.001 |

Note: *SE*, standard error. Trajectory 0 is named "low- fluctuation frailty," Trajectory 1 is named "high-improvement frailty," Trajectory 2 is named "high- deterioration frailty."


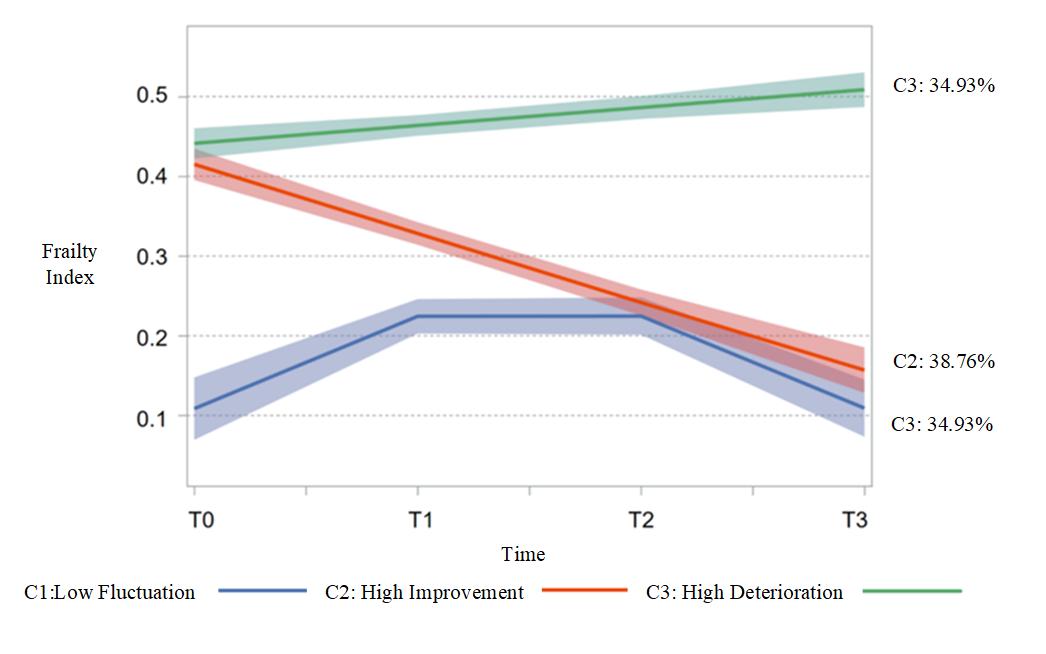


**Figure S2 Trajectories of the frailty index**
