# Supplementary material for: Predicting Frailty Trajectories Using Interpretable Machine Learning Among Older Adults Following Hip Surgery: Prospective Longitudinal Study
Source: JMIR Aging. 2026 Jun 16;9:e90705. doi: 10.2196/90705 (PMC13271583; doi:10.2196/90705)
Supplement: Multimedia Appendix 4 [file aging-v9-e90705-s004.docx]

**Table S6 Variables and their coefficients identified through LASSO regression**

| **Variables** | **Coefficient** |
| --- | --- |
| Age | 0.00430 |
| Gender | 0.03901 |
| Live arrangement | -0.08743 |
| Albumin | -0.02008 |
| hsCRP | -0.21736 |
| Drinking | 0.00557 |
| Teeth number | -0.05541 |
| Comorbidities number | -0.11674 |
| Activities of daily living | -0.06183 |
| Nutritional status | -0.08403 |
| Social support level | -0.00161 |
| Living environment score | -0.04209 |

**Table S7 Multicollinearity diagnostic of predictor variables**

| **Variable** | **Unstandardized Coefficients B** | **Standardized Coefficients Std. Error** | **Standardized Coefficients Beta** | **t** | **Sig.** | **Collinearity Statistics Tolerance** | **VIF** |
| --- | --- | --- | --- | --- | --- | --- | --- |
| Constant | 4.480 | .522 |  | 8.574 | .000 |  |  |
| Age | .007 | .004 | .078 | 1.691 | .093 | .642 | 1.558 |
| Gender | .164 | .064 | .098 | 2.570 | .011 | .933 | 1.072 |
| Live arrangement | -.100 | .085 | -.051 | -1.166 | .245 | .708 | 1.412 |
| Albumin | -.049 | .069 | -.030 | -.715 | .476 | .772 | 1.295 |
| hsCRP | -.285 | .066 | -.178 | -4.319 | .000 | .796 | 1.257 |
| Drinking | .086 | .064 | .052 | 1.344 | .180 | .903 | 1.107 |
| Teeth number | -.052 | .006 | -.440 | -9.282 | .000 | .600 | 1.667 |
| Comorbidities number | -.241 | .061 | -.155 | -3.941 | .000 | .874 | 1.144 |
| Activities of daily living | -.107 | .046 | -.098 | -2.325 | .021 | .765 | 1.308 |
| Nutritional status | -.134 | .042 | -.130 | -3.214 | .002 | .821 | 1.218 |
| Social support level | -.010 | .005 | -.080 | -1.959 | .052 | .800 | 1.251 |
| Living environment score | -.055 | .013 | -.177 | -4.352 | .000 | .818 | 1.222 |
